# Isolation of *Brucella abortus* biovar 1 from human lumbar disc bulging: a case report of brucellar discitis

**DOI:** 10.1186/s12879-021-06538-1

**Published:** 2021-08-19

**Authors:** Saeed Alamian, Afshar Etemadi, Mohammad Reza Samiee, Maryam Dadar

**Affiliations:** 1 Razi Vaccine and Serum Research Institute, Agricultural Research, Education and Extension Organization (AREEO), Karaj, Iran; 2 Arad Medical and Molecular Laboratory, Karaj, Iran

**Keywords:** *Brucella abortus*, Lumbar disc, Bulging infection, Magnetic resonance imaging

## Abstract

**Background:**

Brucellosis is an endemic zoonotic disease with rising health and economic concerns in many areas worldwide. Musculoskeletal pains are among the main complications of human brucellosis, which are often difficult to diagnose due to the variability of clinical symptoms. Brucellar discitis is a very disabling problem in some chronic forms of the disease which may lead to serious vertebral and neurological consequences.

**Case presentation:**

In this case report, we reported the isolation of *Brucella abortus* from lumbar disc bulging in a woman who had rheumatoid arthritis and diabetes mellitus as underlying conditions. The patient had several negative brucellosis serological tests and dorsolumbar pains with urinary incontinence over a 2-month period. The diagnosis was confirmed by magnetic resonance imaging (MRI) examination of lumbar spine as well as disc culture. MRI examination was performed without intravenous contrast and revealed the presence of disc bulging, left foraminal narrowing at L5-S1, left foraminal narrowing, anterolisthesis grade II at L4-L5. The diagnosis was also confirmed by isolation of *B. abortus* biovar 1 from bulging disc culture. The isolate was characterized by AMOS PCR, Bruce-ladder PCR and biotyping, resulting in the identification of *B. abortus* from L4-L5 and L5-S1 disc bulging regions. The patient was treated with two drugs i.e. doxycycline and rifampin for 3 months. In the follow-up, in addition to improving the patient’s general condition, low-back pain was also significantly reduced.

**Conclusions:**

MRI, serology, cultural and molecular test along with patient history are important to make a rapid diagnosis of brucellosis’ discitis, thereby decreasing the delay for the brucellosis treatment. The present report suggests that the infection by *Brucella* spp. should be fundamentally considered among the causative agents of back pain especially in the endemic areas of *Brucella* infections.

## Background

Brucellosis is a zoonosis with a world-wide distribution, most commonly encountered in the Middle East, Latin America and Mediterranean countries. The disease is a common bacterial zoonotic infection between humans and animals, affecting various organs of the body and leading to a wide range of clinical manifestations [1, 2]. The most common clinical manifestations of human brucellosis are fever, chills, sweating, fatigue, headache, as well as musculoskeletal and joint pain. Moreover, lumbar area is the most commonly affected region in spinal brucellosis and represents one of the most important complications in human brucellosis [3–5]. Furthermore, brucellar discitis could occur through the intervertebral disc without spondylitis [6, 7]. Beside the development of chronic back pains, sciatica, disc bulging and disc herniation can be reported in patients with discitis [8]. Here, we report the first isolation of *Brucella abortus* from a disc bulging infection with brucellar discitis in Iran, involving the lumbar and sacrum spine regions simultaneously. The patient had no close contact with animals, travel history, or consumption of unpasteurized dairy products.

## Case presentation

**Fig. 1:**
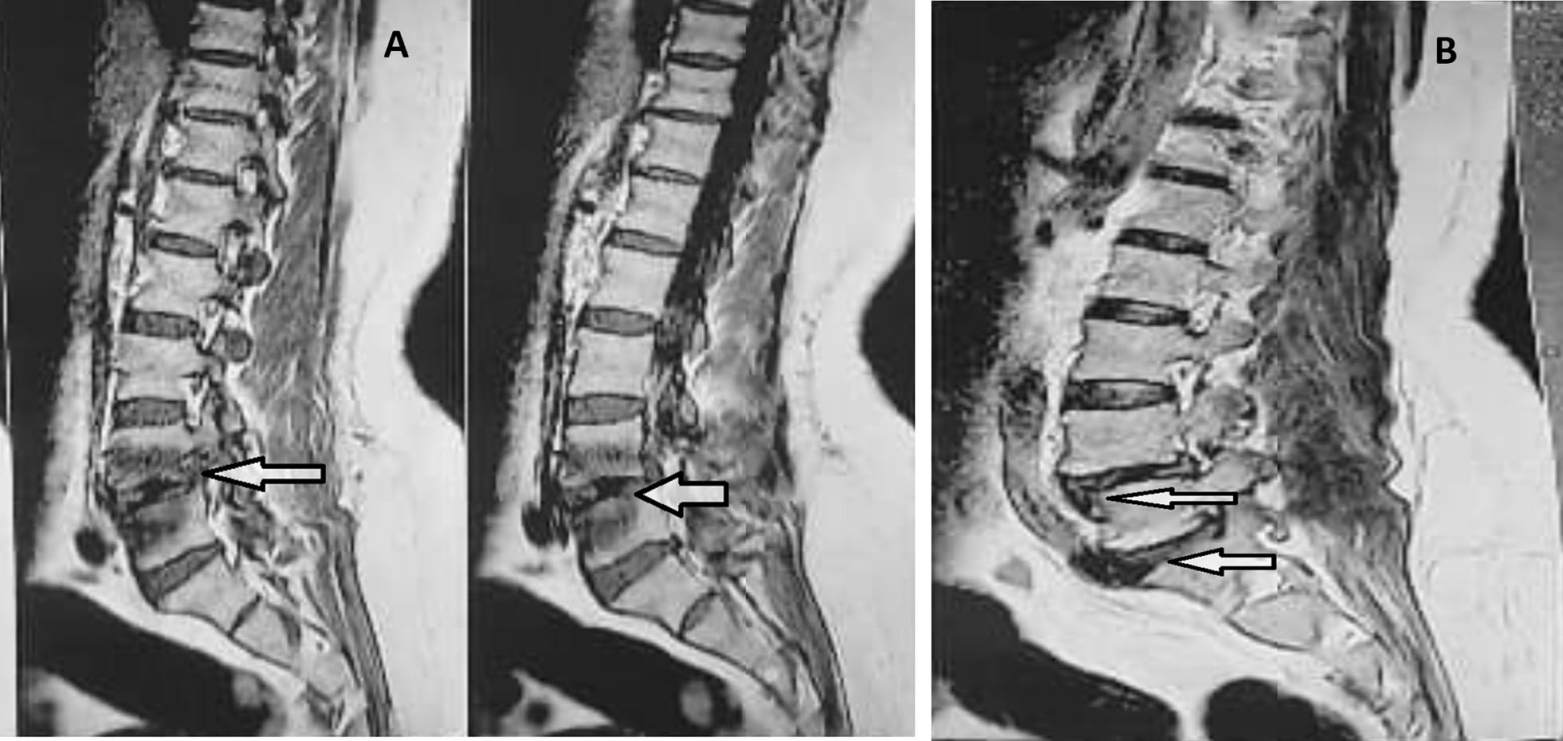
**A** MRI depicting the discitis involvement around L4-L5, **B** discitis involvement around L4-L5 (Up arrow) and discitis involvement around L5/S1 (Down arrow)

In December 2020, a 48-year-old housekeeping female was admitted to the Alborz Hospital (Western Iran) due to dorsolumbar pains for over 2 months and fever (the temperature of 37.8–38). She had urinary incontinence during 1 month and declared no close contact with animals, recent travel history, or consumption of unpasteurized dairy products. It also suffered from type 2 diabetes mellitus and rheumatoid arthritis as underlying conditions. She complained of longstanding pain in the back, and lower-back regions. She reported pain and tenderness in the lower back that led to restrictions on back movements. The patient had several negative brucellosis serological tests including Rose Bengal test (RBT), Serum Agglutination test (SAT) and 2-Mercaptoethanol (2ME). For further evaluation, lumbosacral magnetic resonance imaging (MRI) was performed and brucellosis was suspected through the involvement of the lumbar disc. The MRI diagnosis of lumbar spine without intravenous contrast showed that patient had disc bulging, left foraminal narrowing at L5-S1, left foraminal narrowing, anterolisthesis grade II at L4-L5 (Fig. 1A and B).

**Fig. 2:**
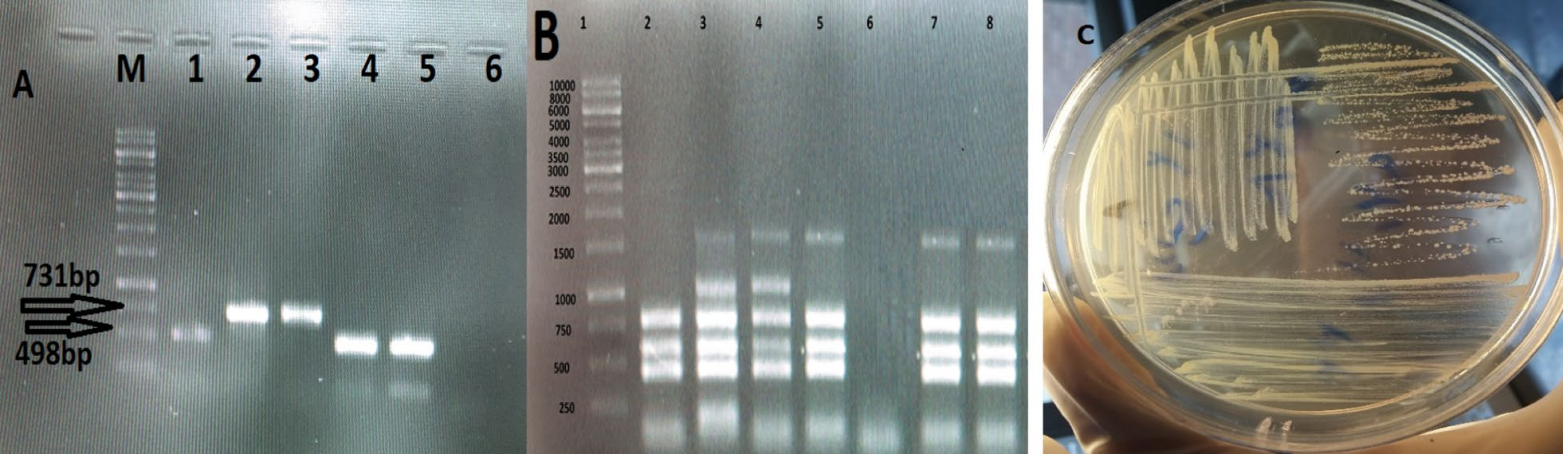
**A** agarose gel electrophoresis (1%) of PCR amplified AMOS- PCR products from isolated bacteria samples. Lane M indicated DNA marker (1000 bp DNA ladder). Lane 1 shows reference bacteria of *B. abortus* 544; Lane 2 shows reference bacteria of *B. melitensis* 16 M; Lane 3 corresponds to wild type of B. *melitensis* ; Lane 4 and 5 shows *B. abortus* from extracted DNA of isolated bacteria from disc culture. **B** agarose gel electrophoresis (1%) of PCR amplified Bruce-ladder PCR products from DNA bacterial samples. Lane 1 shows DNA marker (1000 bp DNA ladder). Lane 2 corresponds to reference bacteria of *B. abortus* RB51; Lane 3 shows reference bacteria of *B. melitensis* Rev1; Lane 4 shows reference bacteria of *B. melitensis* 16 M; Lane 5 illustrates reference bacteria of *B. abortus* 544; Lane 6 negative control, Lane 7 and 8 corresponds to isolated of isolated bacteria from disc culture. **C** colony of *B. abortus* on selective medium

However, the lumbar spine was normal in shape and alignment. The vertebral bodies marrow signal intensity and disc space height seemed grossly normal. There was no evidence of canal stenosis and no signal abnormality was observed in spinal cord including conus medullaris. Moreover, the pelvic MRI showed adequately distended urinary bladder with normal wall thickness. No apparent lymphadenophathy and abnormality on soft tissue were visible. The disc tissue sample was collected according to the standard operating practices for elective lumbar microdiscectomy [9]. Disc specimen was aseptically placed into a closed sterile micro-tube to minimize the risks of post-resection contaminations. The size of the resected disc specimen was 3 × 3 × 5 mm. The disc fragment was homogenized by mortar under aseptic conditions and placed into a pestle containing 3 ml of *Brucella* broth medium for culture (Himedia, India). A total of 200 µl of the resultant homogenate was inoculated on to *Brucella* selective supplement medium containing the cycloheximide (50 mg), vancomycin (10 mg), polymyxin B (2500 IU), nalidixic acid (2.5 mg), nystatin (50,000 IU), bacitracin (12,500 IU) (Oxoid, UK) along with 5% inactivated horse serum. The inoculated plates of bulging disc specimen were incubated under appropriate protection with 10% CO_2_ at 37 °C for 10 days. Typical isolated *Brucella* colonies were identified by conventional and molecular biotyping analyses. Common specific phenotypic features of *Brucella* spp. were observed in the isolated bacteria i.e. Gram negative bacteria with shiny and smooth surface exhibiting a translucent honey color (Fig. 2C). The *Brucella* isolate was identified as *B. abortus* biovar 1 using conventional biotyping methods [10]. Accordingly, the isolate was identified as wild type *B. abortus* using Bruce-ladder and AMOS PCR. AMOS PCR results showed a 498 bp *B. abortus* specific band, common to biovars 1, 2 and 4 [11]. This result was further confirmed by the Bruce-ladder PCR resulting to PCR products of 1682, 794, 587, 450 and 152 bp in size (Fig. 2A and B). The patient was treated with two drugs i.e. doxycycline 100 mg plus rifampin 300 mg every 12 h for 3 months. In the course of the follow-up, the patient’s general condition has remarkably improved and the low-back pain was reduced. After a 3-month treatment, the urinary incontinence had recovered well. However, we were not able to follow the patient after 3 months as she did not appear at the next checkup for further tests due to COVID-19 strict mobility restrictions and the improvement of her low-back pain symptoms.

## Discussion and conclusions

The skeletal complications following brucellosis are very common in endemic regions, including Iran [2]. Therefore, manifestations such as musculoskeletal pain, osteoarthritis, sacroiliitis, large joint involvement, spondylitis and discitis are expected. Previous studies showed that *Brucella melitensis* is the main species responsible for human brucellosis, thereby representing an important public health issue in many regions worldwide [5, 12]. However, in this case we isolated the *B. abortus* from bulging disc infection for the first time, giving us an important overview on the *Brucella* species susceptible to induce this form of infection. This result was also of overwhelming importance in improving patient outcomes as this case showed negative serological tests despite the isolation of *B. abortus*, thereby indicating lack of specific *Brucella* antibody titers in certain patients suffering from focal complications. Similarly, two cases of brucellar spondylodiscitis of the lumbar area showed negative serological tests despite positive *Brucella* spp. culture and typical radiological alterations [13, 14]. Therefore, negative results of serology could not exclude the presence of focal complications in patients suffering from brucellosis in the chronic course of the disease.

Different antibiotics such as doxycycline, rifampicin, trimetroprim, sulphamethoxazole objective, ciprofloxacin, and aminoglycoside as well as their combinations are proposed in the spinal brucellosis treatment [15]. In our case, appropriate antibiotic treatment for 12 weeks led to a significant improvement of the patient’s conditions. Several combinations of antibiotics could be used in the treatment *Brucella* infection and these regimens often include doxycycline and rifampin with or without an aminoglycoside [15]. However, complicated cases received antibiotics for a longer period, as shown in the present case where antibiotic regime was extended for 8 months. It is important for clinicians to be aware of this disease and consider it in their differential diagnosis for patients with unexplained musculoskeletal symptoms in conjunction with fevers. Although the isolation of *B. abortus* is rare in human brucellosis, this pathogen could induce complication in joints and bones such as tenosynovitis, arthritis, sacroiliitis, bursitis, osteomyelitis, and spondylitis [16]. However, our case showed a discitis affecting the intervertebral disc in two regions. Humans could be infected with *B. abortus* by ingesting contaminated dairy products or via contaminated mucous membranes (including the respiratory and conjunctiva tract), abraded skin, inhaling airborne agents, or very rarely, the bacteria may spread from person to person through sexual contact [17]. However, in the present case, the patients did not remember any relevant sources of infection. Finally, a multi-disciplinary approach is hotly demanded to manage a complex spinal brucellosis infection. In this respect, it is very important to differentiate tuberculosis-induced spondylodiscitis from brucellosis spondylodiscitis due to similar radiological manifestations. For this purpose, culture, PCR and serological testing are very helpful methods. A history of contact with livestock and their products is a diagnostic guide for brucellosis. The diagnosis of brucellosis is mainly based on the patient clinical history and epidemiology. Due to the endemic nature of brucellosis in Iran, physicians should consider brucellosis discitis among the differential diagnoses in patients with long time back pain. Initial management should consist of prolonged and aggressive antibiotic regime. By early diagnosis and treatment, the patient’s pain and discomfort could be relieved, thereby preventing complications such as paravertebral abscess and neurological manifestations. Although, serologic tests are very useful in brucellosis diagnosis, but it should be highlighted that negative serologic tests could not exclude the diagnosis of brucellosis in endemic area. Thus, the specificity of serological assays is low for predicting accurately the course of the disease, since titers may remain positive after the resolution of symptoms or negative in the case of the focal persistence of the disease.

One of the preferred diagnostic methods for spinal cord involvement in brucellosis is MRI, despite its low specificity for definitive diagnosis of osteoarticular lesions. But in endemic areas such as Iran, it is highly recommended to perform an MRI for patients with suspected brucellosis with evidence of long back pain. In certain cases, surgery should be considered if there is a poor response to medical management. However, it should be noted that the diagnosis and treatment of Brucellar discitis remain very difficult and required the support of specialized reference laboratories.

## Data Availability

The data used in this study will be available from the corresponding author.

## Abbreviations

MRI: Magnetic resonance imaging
RBT: Rose Bengal test
SAT: Serum Agglutination Test
2ME: 2-Mercaptoethanol
